# Fibroblast-myofibroblast crosstalk after exposure to mesenchymal stem cells secretome 

**Published:** 2018

**Authors:** Khadijeh Jalili Angourani, Sogol Mazhari, Shirin Farivar, Donya Salman Mahini, Abdolreza Rouintan, Kaveh Baghaei

**Affiliations:** 1 *Department of Molecular and Cell Biology, Faculty of Life Sciences and Biotechnology, Shahid Beheshti University, Tehran, Iran *; 2 *Basic and Molecular Epidemiology of Gastrointestinal Disorders Research Center, Research Institute for Gastroenterology and Liver Diseases, Shahid Beheshti University of Medical Science, Tehran, Iran.*; 3 *Gastroenterology and Liver Diseases Research Center, Research Institute for Gastroenterology and Liver Diseases, Shahid Beheshti University of Medical Sciences, Tehran, Iran*; 4 *Department of Plastic and Reconstructive Surgery, Shahid Beheshti University of Medical Sciences, 15 Khordad Hospital, Tehran, Iran*

**Keywords:** MSCs-secretome, Fibroblast, Myofibroblast, Alpha-smooth muscle actin, Collagen I, Collagen III

## Abstract

**Aim:**

The aim of the present study was to investigate the effect of human bone marrow-derived mesenchymal stem cells conditioned medium on fibroblast to myofibroblast differentiation.

**Background:**

Mesenchymal stem cells have a long-term clinical application and widely have used in autoimmune disease and regenerative medicine. However, some MSCs derived cytokines such as TGF-β could have a dual role in suppression or progression of disease. Fibroblast activation and extracellular matrix production are two key features of wound healing which mostly are controlled with multifunctional cytokine TGF-β1.

**Methods:**

Bone marrow MSCs were isolated, cultured and used for conditioned medium preparation. The flow cytometry analysis was done for MSCs cell surface markers. MRC-5 subconfluent cells were starved with the medium containing 0.5 % FBS for 24h, then treated with exogenous TGF-β1 (10ng/ml as positive control) and MSCs-conditioned medium for 48h. Finally, the mRNA expression of three target genes: collagen I, collagen III and α-SMA were evaluated by RT-PCR technique.

**Results:**

Our findings demonstrated that bone marrow-derived mesenchymal stem cells-conditioned medium (secretome) significantly upregulated type I and III collagen expression but non-significantly α-SMA gene expression.

**Conclusion:**

Totally, Real Time PCR results suggest that MSCs conditioned medium activates differentiation of fibroblast to myofibroblast phenotype as confirmed through the presence of α-SMA, collagen I and collagen III expression compared to control in MRC 5 cells.

## Introduction

 Fibrosis is a heterogeneous connective tissue disorders, characterized by aberrant and the excessive deposition of extracellular matrix (ECM) components (1).

Despite the etiology, fibrotic disease affects most tissues including: skin (2), liver, lung (3) and kidney (4). Understanding the molecular and cellular mechanisms leading to fibrotic statues is so important because the development of fibrotic process, in some cases, leads to organism failure and increase mortality rate (5). Fibroblasts and myofibroblasts, as main effector cells involved in fibrosis, are responsible for producing extracellular matrix proteins such as collagens (types I and III), cellular fibronectin (6), alpha-smooth muscle actin(α-SMA)(7) and other elements (8). The switch between fibroblast to myofibroblast phenotype, the phenomenon occurred in tissue repair, is more influenced by transforming growth factor-β1(TGFβ 1), which is produced and secreted by inflammatory and effector cells in inflammatory environment. Multi-functional cytokine TGF-β1 modulates many biological processes such as differentiation and remodeling of extracellular matrix components (9). TGF-β1 stimulates expression of pro-fibrotic genes and components of the myofibroblast contractile cytoskeleton and other component of ECM (10), which are necessary and vital for wound contraction and tissue repair (11).

Nowadays, implanting mesenchymal stem cells (MSC) to fix tissue homeostasis in impaired organs through resolution of fibrosis. have raised considerable interest (12). MSCs for therapeutic purposes can be easily aspirates from different tissues including: adipose (13), umbilical cord (14) and most from bone marrow (15). MSCs are characterized by positive mesenchymal cell surface markers such as CD73, CD90 and CD105 and negative markers: CD 14, CD34 and CD45. They are capable of self-renewing and differentiating towards several mesodermal and none-mesenchymal lineages (16). Pluripotent bone marrow-derived mesenchymal stem cells (BMSCs) aspirates from bone marrow easily and they are capable of self-renewing and differentiation to mesodermal lineage such as adipocytes, cardiomyocytes (17) and non-mesodermal lineage such as neurons (18). 

Many clinical trials have utilized MSCs for regenerative medicine applications in order to restoration of damaged tissues and treat fibrotic conditions (19, 20). BM-MSCs, have been known to produce and secrete factors such as TGF-β1 (21) which promotes the differentiation of fibroblast cells. The efficacy of bone marrow MSCs in reduction of organ injury in disease model have been shown in some studies (22, 23). Subsequently, some studies represented that MSCs-derived conditioned medium may be related to observed therapeutic impact of these cells and they can exert the therapeutic efficacy of MSCs (24).

In this study, we have used MRC5 cell line (human fetal lung fibroblasts) to investigate the impact of BM-MSCs conditioned medium (soluble signaling factors released by BMSCs) on fibroblasts activation and cell differentiation. 

## Methods

**Fibroblast culture**

MRC5 lung fibroblasts (human lung fibroblasts), a gift from Tehran university, were cultured in Dulbecco's Modified Eagle's medium (DMEM) with 10% fetal bovine serum (FBS) supplemented with 1% Lglutamine and 1% penicillin/streptomycin solution. Cells were incubated at 37˚C in 5% CO_2 _and passaged upon reaching 80% confluency, by 0.025% trypsin/EDTA (Gibco-USA). All experiments in this study were performed on low passage number cells (between passages 2-5).

**MSC isolation and expansion**

MSCs were isolated from human bone-marrow following previously described methodology by our colleague’s work. (25) Briefly, human whole bone marrow (obtained from iliac crest) was collected into K2EDTA tube. The separated buffy coat was suspended in 1.5 mL PBS and layered onto equal volume of Ficoll (GE health care, USA) and centrifuged (400 × g, 20 min). Cells at the interface were removed, washed twice in PBS and then cultured on T-75 flasks in DMEM medium consisting of 10% FBS, 1% penicillin/streptomycin ( Gibco-Invitrogen, Carlsbad, USA; respectively) and incubated at 37°C, 5% CO2 and 95% atmosphrer. After about 80% confluent cells were passaged and then used for conditioned medium preparation. 

**Flow Cytometric Characterization of MSCs**

To validate cells as MSCs, we have done flow cytometry assay. Low passage number cells were harvested and resuspended in 1% Bovine Serum Albumin (BSA) and were stained with antibodies against CD14, CD34, CD45, CD90, CD105 and CD73 (PE) (ebioscience, Germany). Unstained cells used as control. After 15 minutes remaining in 4 °C, antibodies were neutralized with buffer staining and centrifuged for 10 minutes at 4 °C, 1500 rpm. CD markers were detected by FACS flow cytometry and Cell Quest Software (Becton Dickinson, UK). Flowing software2 were used for final analyzing. 

**Table 1:** Primer sequences of genes used in the present study

Gene name	Primer sequence	Product size	Gene bank ID
Col 1	F: 5’ CCCCAGCCACAAAGAGTCTA 3’ R: 5’ TGGTGGGATGTCTTCGTCTT 3’	149	NM 000088.3
Col III	F: 5’ TGGAAACTGGGGAAACATGC 3’ R: 5’ GGATTGCCGTAGCTAAACTGA 3’	142	NM 000090.3
α -SMA	F: 5 ’AGACGGGAATCCTGTGAAGC 3’ R: 5’ TGTCCCATTCCCACCATCAC 3’	182	NM 001141945.2
GAPDH	F: 5’ GAAGGTGAAGGTCGGAGTCA 3’ R:5’ AATGAAGGGGTCATTGATCA 3’	145	NM001256799.2

**Figure 1:**
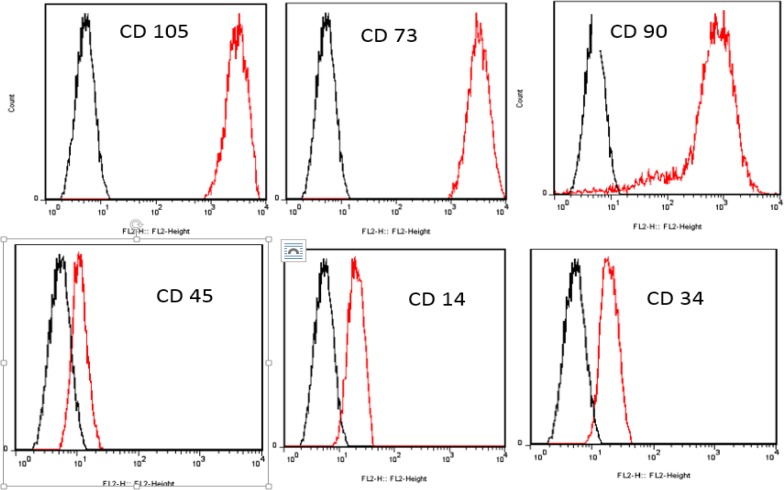
Characterization of cell surface markers of MSCs by flow cytometry**. **Flow cytometry analysis of cell surface markers on cells confirmed them as MSCs, which were positive for CD73, CD90, and CD105 markers and negative for CD14, CD34 and CD45. The black plots presented as unstained control and the red one as MSCs markers

**Figure 2:**
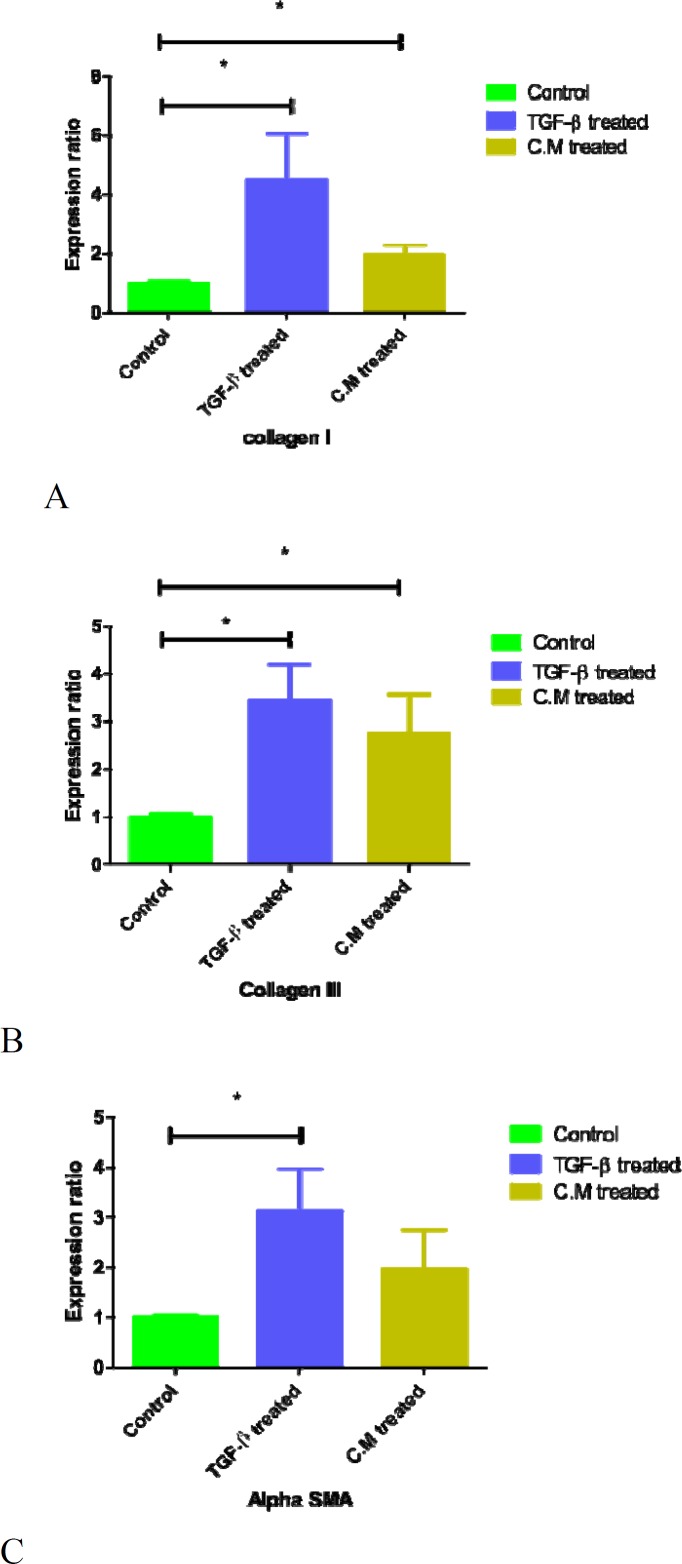
Effect of BMMSCs-conditioned medium and TGF-β1 on collagen I, III and α-SMA mRNA expression. Subconfluent serum starved cells were treated with either TGF- β1 (10ng/ml) or BM-derived MSCs conditioned medium for 48 hours. qRT-PCR for A) Col1a, B) Col 3a and C) α-SMA mRNA expression in MRC-5 cells showed increased production of collagen I, III and α-SMA when compared with control group. BM: bone marrow CM: conditioned medium, TGF-β1, transforming growth factor-β1, α-SMA: Alpha smooth muscle actin.

**Conditioned Medium from human MSCs preparation**

To prepare CM, low passage number (2-4) cells were seeded into T-75 culture flask in the medium containing DMEM ,10% FBS, 1% penicillin/streptomycin and incubated at 37 °C in a humidified and 5% CO_2_ allowed to reach 80% confluency. In order to get 3% FBS conditioned medium, we tried to decrease the FBS of the medium step by step (7%, 5% and finally 3%every 72h). Cell detachment was achieved by incubation with %0.025 Trypsin/EDTA for at least 5 min at 37 °C. The supernatants from flasks were then collected and centrifuged at 1500 rpm, 4 °C for 12 minutes to remove cellular debris and stored at -80 ° C until use.

**Induction of myofibroblast differentiation by TGF-β1 and MSCs-Conditioned Media**

The *in vitro* differentiation of fibroblast to myofibroblast was achieved using treatment with profibrogenic cytokine TGF-β1 and human MSCs-CM. For this study MRC-5 cells were divided into three groups: control, TGF-β1 treated and conditioned media treated groups. Cells were seeded at density of 75 x10 ^3 ^cells per well, in 6-well plates fed with MRC-5 media (2 ml) and incubated at 37 °C with 5% CO2 and 95% atmosphere, allowed to attach overnight. Subsequently, to induce cell differentiation, cells were growth arrested with serum starvation, so the medium of both treated cells was displaced by medium supplemented with 0.5% FBS and incubated for another 24 hours. On the third day, 10ng/ml of TGF-β1 was added to one of the 0.5% experimental group, whereas the medium of the other experimental group was changed with MSC-CM (in the ratio of 70 % CM and 30% DMEM 0.5% FBS). After 48 hours of treating with TGF-β1(10ng/ml) and CM, the cells were collected and used for RNA extraction.

**Quantitative RT-PCR (qRT-PCR)**

Total RNA of samples were extracted by using RNeasy Mini Kit (Favorgen, Taiwan) based on manufacturer’s instructions and DNA contamination was eliminated via treating samples with 0.5 µl DNase (Thermo Fisher Scientific, USA). Purity of RNA was measured with the Nanodrop device (Thermo Fisher Scientific, USA). Extracted RNA stored at -70 °C until further analysis. Isolated RNA was reverse-transcribed to cDNA (Thermo Fisher Scientific kit, USA) by using random hexamers. To perform Real Time PCR, the primers for target and internal control genes, were designed by primer 3 software and blasted at NCBI (shown in table 1). Gene runner (ver.6.0.04) was used to validate the accuracy and specificity of the primers. PCR reactions were done in duplicate on Rotor Gene Q Series (Qiagen, Germany) and SYBR Green Mastermix (Applied Biosystems) in a final volume of 20 ml containing 2 µl of reverse transcribed cDNA and 0.8 µl specific primers. Finally, the relative expression of target genes were evaluated with the REST 2009 software version 2.0.13 by using human GAPDH for normalization.

**Statistical analysis**

Data are representative as three independent experiments. GraphPad Prism 5 software was used for statistical analysis (t- test) with a probability (P) ≤ 0.05 considered as significant

## Results

**Flow Cytometric Characterization of MSCs**

 Flow cytometery assay along with osteogenic and adipogenic differentiation assays (data not shown) identified cells as MSCs with purity of 98%. Because we have used the same source of MSCs as used in our previous study (25) this time we just considered CD markers again to confirm the presence of MSCs. MSCs exhibited CD73, CD90 and CD105 markers and they lacked CD14, CD34 and CD 45 markers (Figure 1). **BM-derived MSCs-conditioned medium and TGF-β1 effects on expression of col I **

 To investigate MSCs-conditioned medium and exogenous TGF-β(10ng/ml) effects on the mRNA expression of collagen I gene, we performed q RT-PCR on type I collagen (encoded by Col1a 1) in MRC-5 cells. Expression of Col1a gene was significantly upregulated when MRC-5 cells were cultured in both MSC-conditioned medium and TGF-β1 groups in comparison with control group with standard medium. (Fig. 2A). 

**BMMSCs-conditioned medium and TGF-β effects on expression of col III**

 To investigate MSC-conditioned medium and exogenous TGF-β1 (10ng/ml) effect on the mRNA expression of collagen III gene, we performed q RT-PCR on type III collagen gene (encoded by Col3a 1) in MRC-5 cells. Expression of Col3a gene was significantly upregulated when MRC-5 cells were cultured in MSCs-conditioned medium and TGF-β1 groups in comparison with control group with standard medium. (Fig. 2B) 

**BMMSCs-conditioned medium and TGF-β1 Effects** **on expression of α-SMA **

To investigate MSC-conditioned medium and TGF-β1 effect on α-SMA gene expression, we performed q RT-PCR on α-SMA (encoded by ACTA2) in MRC-5 cells treated with both CM and TGF-β1(10ng/ml). Expression of α-SMA gene was upregulated when MRC-5 cells were cultured in MSC-conditioned medium and exogenous TGF-β1 (10ng/ml) compared to control group with standard medium (Fig.2C). Although, the expression of α-SMA was increased in CM treated group but this increased expression was not significantly because of low repetition. We have expected this upregulation will be significant in high repetition. 

**Comparison of genes expression between TGF-β treated and bone marrow MSC-CM treated cells**

 MSCs-conditioned medium, similar to TGF-β1, induced fibroblast to myofibroblast differentiation. RT analysis after 48 hours treating with CM and TGF-β, obtained from three independent experimental repetitions, showed that mRNA expression of collagen types 1, III and α-SMA were upregulated in both groups in comparison with control. However, the effect of TGF-β was greater than conditioned medium.

## Discussion

COL I, III and α-SMA as three major components of extracellular matrix contributing to the tensile strength of connective tissue ECM (26), have been utilized as broad molecular marker for fibroblast to myofibroblast differentiation (27) (28), which is necessary for wound healing function and tissue repair. 

 The usage of MSCs in cell based therapy and curing disease is related to their multi-linage differentiation ability and their rich secretome. Because when they apply to the target tissues, after homing (29), they can differentiate to desired cells, and ameliorate organ damages. BM-MSCs have known to secret some cytokines including TGF-β1 (30), which is the major modulator of fibroblast differentiation, therefore promoting wound healing (31). The principal reason for regenerative application of these cells in disease treatment, is due to their anti-inflammatory capacity and their wide-ranging immunomodulatory properties (32). Smith *et al.* demonstrated that MSCs secretome leads to the elevation of the fibroblast’s proliferation (33). Furthermore, MSCs mostly have been used in tissue repair including: liver (34) and lung (35). So due to these studies, we investigated the paracrine effects of bone marrow derived MSCs-CM on MRC-5 fibroblasts *in vitro. *PCR analysis (Fig. 2, **3** and **4**) showed that in fibroblasts cultured with CM similar to cell treated with TGF-β1 as a positive control, after 48h expression of myofibroblast markers, the mRNA level of Col I and Col III significantly and α-SMA non-significantly were increased. Because the results in both experiments were similar, it seems that interactions between fibroblast and BMMSCs occur by paracrine mechanisms. Geo *et al.* showed that engraftment of MSCs to a rat model of myocardial infarction blocked types I and III collagen(36). In the other study (37), they used cardiac fibroblast and demonstrated that MSCs reduced collagen I and III expression significantly and their result was contradictory to our result. Their result was in accordance with anti-fibrotic effect of MSCs. Also the other study showed that BMMSC conditioned medium increased fibroblast proliferation and stimulated fibroblast migration (38). Altogether, the analysis of our study suggests secreted factors present in bone marrow-derived MSCs conditioned medium exhibited an influence in inducing elevation of myofibroblastic markers on MRC-5 cells limited. This limit upregulation of target genes is beneficial for wound healing process and disease in which there is aberrant inflammatory response. 

According to the present study’s results in compatible with the others, MSCs conditioned medium are suitable candidates for use in cell-free based therapy for wound treatment because they have made differentiation of fibroblast to myofibroblast at least in part through increased production of collagen I, III and α-SMA. These characteristic of MSCs may be advantageous for the treatment of organs failures and can improve tissue repair.
